# ‘*You Can Be Simultaneously Powerful and Marginalised at the Same Time in Different Ways’.* An Intersectional Examination of Barriers and Facilitators of Help‐Seeking for Eating Disorders by People From Under‐Served Groups

**DOI:** 10.1002/erv.70131

**Published:** 2026-05-29

**Authors:** Jessica Wilkins, Lucy Gallagher, Karina L. Allen, Ulrike Schmidt

**Affiliations:** ^1^ Centre for Research in Eating and Weight Disorders Institute of Psychiatry, Psychology and Neuroscience King's College London London UK; ^2^ South London and Maudsley NHS Foundation Trust London UK

**Keywords:** eating disorders, help‐seeking, intersectionality, marginalised, under‐served

## Abstract

**Objective:**

To explore barriers and facilitators to help‐seeking by people from under‐served groups in eating disorders (EDs).

**Methods:**

Seventeen participants with lived experience of an ED, identifying as members of groups traditionally under‐served in ED research (ethnic minority, sexual or gender minority, or men), took part in semi‐structured interviews. Data were analysed using reflexive thematic analysis, with inductive coding, interpretive theme development, and deductive mapping onto a micro‐, meso‐, and macro‐level intersectional framework.

**Results:**

Participants identified factors consistent with existing research on barriers and facilitators to help‐seeking alongside factors unique to under‐served groups. These included internalised stigma, stereotypes about EDs, lack of inclusive environments and poor representation in ED resources. Systemic factors comprised barriers to accessibility or strict inclusion criteria for specialist treatment. Facilitators to help‐seeking included inclusive care, and the utility of information seeking online.

**Conclusion:**

Findings extend existing evidence on ED help‐seeking and offer novel insights into experiences of under‐served groups in ED. To improve help‐seeking among under‐served groups, recommendations include interventions to address stigma, challenging stereotypes about EDs, and ensuring inclusive representation in psychoeducation and service provision. At a systemic level, reforms to service commissioning and inclusion criteria are necessary to create equitable access to care.

## Highlights

- We conducted qualitative interviews with participants from traditionally under‐served groups in eating disorders (EDs), identifying barriers and facilitators to help‐seeking.
- The findings support existing research on barriers and facilitators to help‐seeking for EDs, while offering novel insights into the distinctive experiences of individuals with marginalised social characteristics.
- Recommendations are made for interventions to improve help‐seeking for ED by people from under‐served groups including targeted awareness building for individuals and health care professionals, practical considerations for services and lowering barriers to accessing specialist services.

## 1 Introduction

Eating disorders (EDs) are serious mental illnesses characterised by persistent disturbances in eating behaviours and related cognitions. They present a significant public health concern due to their high morbidity, mortality, and chronicity (Schmidt et al. 2025). Furthermore, EDs carry profound social and economic consequences, including impaired psychosocial functioning, reduced quality of life, and substantial healthcare burden (Liu et al. 2025). Early intervention is associated with improved outcomes for EDs (Austin et al. 2022). However, individuals with EDs often wait years before accessing specialist treatment, ranging from an average of 2 years for anorexia nervosa (AN) to up to 6 years for binge eating disorder (BED) (Austin et al. 2021). These delays reflect the substantial difficulties individuals face when seeking help for EDs. Research on barriers to help‐seeking has examined individual factors and systemic‐level factors. Individual barriers include shame, denial of the problem, and lack of awareness of resources (Ali et al. 2016; Radunz et al. 2023). Systemic‐level barriers include lack of access to specialist services and long wait times for treatment (Daugelat et al. 2023). Existing research primarily focuses on the experiences of *cis*‐gender, white women and samples are often already engaged in specialist treatment for EDs. This limits generalisability and overlooks those who never seek or receive care (Ali et al. 2025).

Individuals from under‐served groups may encounter unique barriers to help‐seeking. Individuals from minoritised ethnic backgrounds remain under‐represented in ED research (Egbert et al. 2022), limiting the integration of their experiences into the broader understanding of EDs. For example, despite similar prevalence of BED among Black and White individuals, Black individuals are significantly less likely to seek help (Coffino et al. 2019). Similar disparities exist for sexual and gender minority groups (Heiden‐Rootes et al. 2023; Parker and Harriger 2020), people with larger body sizes (Ralph et al. 2022) and males (Bomben et al. 2022) who report ED symptoms but lower rates of diagnosis and treatment. These groups face distinct systemic challenges in accessing care, such as cultural stigma and under‐recognition of symptoms (Levinson et al. 2024). As a result, individuals from these groups are less likely to access timely and appropriate treatment (Chowbey et al. 2012; Penwell et al. 2024), exacerbating illness severity and widening disparities in treatment outcomes.

### 1.1 Intersectional Theory

Originally described by Crenshaw (1991), intersectionality has no universally‐cited definition but it is generally understood as a framework recognising that inequities emerge from the interaction of multiple social locations within interconnected systems. The term social location emphasises that experiences of marginalisation and privilege arise from these dynamic interactions rather than single characteristics, conferring power in some areas while increasing vulnerability in others (Hankivsky 2014).

Intersectional influences can be organised into interacting micro‐, meso‐, and macro‐levels (Cook et al. 2014; Hankivsky 2014; Rai et al. 2020). Macro‐level factors include systemic factors such as policies, system process, and cultural norms. Meso‐level factors involve communities and interpersonal interactions. Micro‐level factors reflect individual experiences, behaviours, and psychological responses to broader macro‐ and meso‐level factors, including internalised stigma. These interdependent factors are often studied in isolation, overlooking how they interact to create unique and compounded barriers and facilitators to help‐seeking. A multilevel framework rejects the idea that inequalities arise from single factors and has been applied in physical health research to understand how stigma related to health conditions (e.g., HIV, leprosy) interacts with social characteristics (e.g., ethnicity, sexual orientation) to impact help‐seeking and access to care (Logie et al. 2011; Rai et al. 2020). These studies describe how stigma shapes resources, interpersonal interactions, and perceptions of health conditions, thereby influencing help‐seeking. Building on this, intersectional approaches have informed interventions to reduce inequalities and improve access for under‐served groups (Rai et al. 2020).

Qualitative research centring the lived experiences of under‐served groups in EDs remains limited (Egbert et al. 2022) and few studies have examined how intersecting marginalised social locations (e.g., ethnicity, body size) shape help‐seeking behaviour and pathways into care (Wilkins et al. 2025). Existing work, including research with Indigenous communities in New Zealand, highlights barriers such as inconsistent assessment practices and geographically inaccessible services (Clark et al. 2023). Addressing these gaps is critical to improving understanding of help‐seeking and promoting timely access to evidence‐based treatment.

Informed by an intersectional lens, this study aims to advance understanding of barriers and facilitators to ED help‐seeking among under‐served groups, and how participants experienced and understood seeking care.

## 2 Methods

### 2.1 Ethics

Ethical approval for this study was granted by King's College London Research Ethics Committee (Reference: HR/DP‐24/25‐43912).

### 2.2 Sample

Inclusion criteria were developed through stakeholder focus groups including people with lived experience of an ED and clinicians. In line with NIHR INCLUDE guidelines for engaging under‐served groups (NIHR 2020), we collaborated with third‐sector organisations supporting minoritised communities. Inclusive study design features included participant remuneration (£20 each) and flexible response formats (e.g., online, in writing).

Inclusion criteria were: (1) aged 16 and over; (2) living in the United Kingdom (UK); (3) current or past experience of any ED (formal diagnosis or experience of specialist ED treatment not required); and (4) self‐identified as belonging to a sexual/gender minority, ethnic minority, or as a man. All participants gave written informed consent prior to inclusion in the study. Sample size was initially informed by previous studies involving under‐served ED populations (Potterton et al. 2020; Tsong et al. 2023) and during analysis was judged to provide sufficient information power to generate rich accounts of diverse help‐seeking experiences (Malterud et al. 2016).

### 2.3 Procedure

Participants first completed a brief demographic questionnaire, and the Eating Disorder Examination Questionnaire (EDE‐Q) via Qualtrics (qualtrics.kcl.ac.uk). The EDE‐Q is a 28‐day self‐report measure of ED thoughts and behaviours, with established validity in sexual and gender minority groups and in men (Klimek et al. 2021; Scharmer et al. 2020). Scores greater than 1.6–2.8 (depending on population and norms) are considered clinically meaningful. Although not diagnostic, higher EDE‐Q global scores indicate greater overall symptom severity (Carey et al. 2019).

After completing the questionnaires, participants took part in a semi‐structured interview about their help‐seeking experiences (see Supporting Information S1). Recruitment and interviews occurred between October 2024 and May 2025. Interviews (45–90 min) were conducted one‐to‐one online via Microsoft Teams by JW. With consent, they were audio‐ and video‐recorded, transcribed verbatim (capturing spoken words only), anonymised, and deleted after transcription. Transcripts were not returned, but participants reviewed the manuscript to confirm accurate representation of their experiences.

To support reflection on participants' social locations, the ‘Wheel of Power and Privilege’ was adapted for the UK context (Duckworth and Hankivsky n.d.). Our Wheel of Social Locations (Figure 1) was used as a visual prompt to facilitate discussion around experiences of marginalisation or privilege across demographic and social characteristics listed on the wheel; it was not included in formal analysis.

**FIGURE 1:**
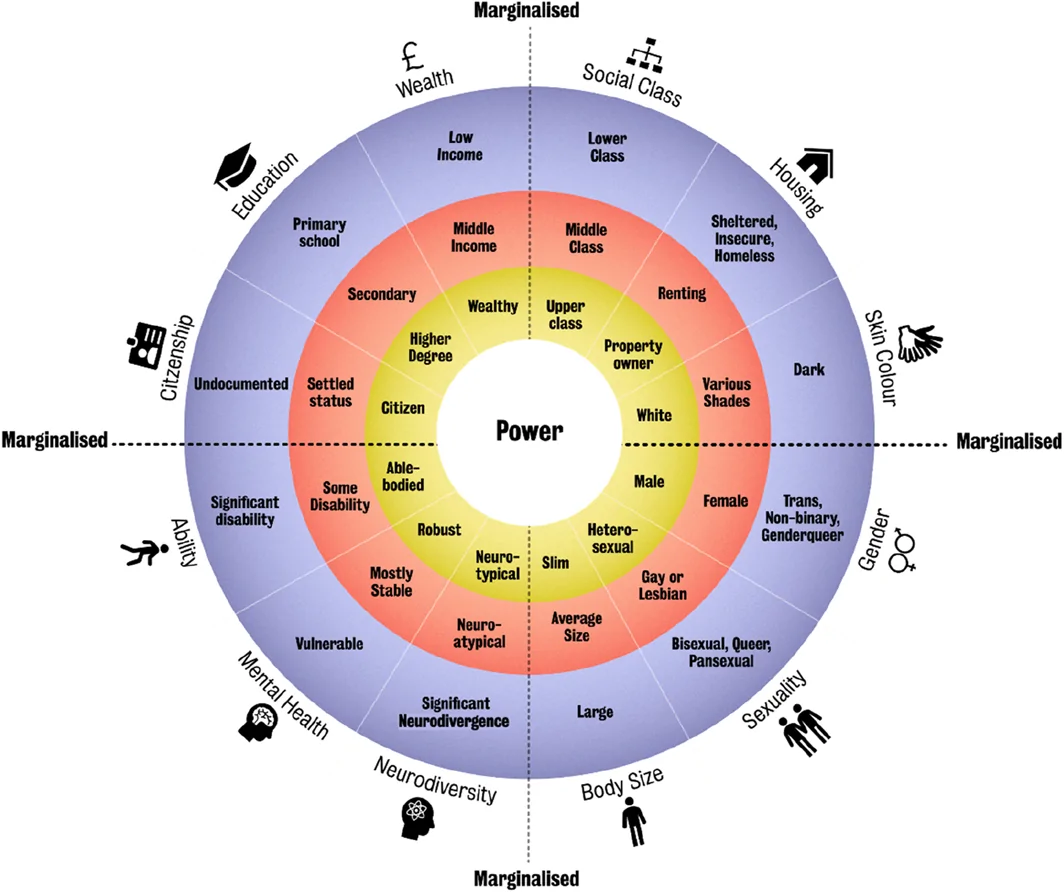
Wheel of social locations. This figure has been adapted from ‘The Wheel of Power/Privilege’ authored by Duckworth and Hankivsky to include marginalised characteristics which may impact the experience of people in the UK experiencing EDs. Proximity to the centre indicates greater societal power, whereas positions further from the centre represent increased likelihood of experiencing marginalisation.

### 2.4 Data Analysis

Transcripts were analysed using Atlas.ti by two researchers (JW and LG). All members of the research team are White, cisgender women working in ED research in the UK. JW and KA are working clinically as psychologists and US is a psychiatrist. Transcripts were coded independently, then discussed in weekly meetings. Reflexivity was supported through individual note‐taking and regular team discussions, including monthly meetings with the wider team. Coding differences were explored through dialogue to deepen interpretation rather than to reach consensus. Analysis followed Braun and Clarke's (2022) six‐phase approach to thematic analysis: data familiarisation, initial code generation, theme identification, theme review, theme definition, and report writing.

Guided by a critical realist framework, analysis adopted an iterative approach combining inductive and deductive coding to explore themes and causal mechanisms in help‐seeking accounts. After developing initial themes we noted their alignment with micro‐, meso‐, and macro‐level intersectionality frameworks [for example, (Ali et al. 2016; Simpson 2009)]. We then drew on this framework as an analytic lens to further interpret and organise the data, in keeping with Braun & Clarke's inductive‐deductive approach (Braun and Clarke 2022). Themes were checked against the verbatim transcripts for accuracy.

## 3 Results

### 3.1 Participant Characteristics

Seventeen participants were included and their demographic and clinical characteristics are detailed in Table 1. 94% (*n* = 16) of participants reported experiencing more than one marginalised social location using the wheel of social locations. Beyond our inclusion criteria, 59% (*n* = 10) self‐reported neurodivergence (Autism or Attention Deficit/Hyperactivity Disorder) and 47% (*n* = 8) a comorbid mental health condition (in addition to an ED). We acknowledge that participants often occupied multiple marginalised social locations; however, given the sample size and variability in identity combinations, we did not undertake further analysis of specific intersections between identities and themes.

**TABLE 1:** Summary of participant demographics (*N* = 17).

Gender	Female	7 (41%)
	Male	6 (35%)
	Transgender/transmasculine	4 (24%)
Sexual orientation	Asexual	1 (6%)
	Gay/lesbian	4 (23%)
	Heterosexual	6 (35%)
	Pansexual	4 (23%)
	Queer	2 (11%)
Ethnicity	Ethnic minority background	6 (35%)
	White	11 (65%)
Diagnosis	AN	8 (47%)
	ARFID	4 (24%)
	BED	4 (24%)
	BN	2 (11%)
	OSFED	2 (11%)
Age	23–57 years	Median = 32
EDE‐Q	*M* = 2.81, SD = 1.31	Median = 3.1

*Note:* N.B. Some participants reported more than one ED diagnosis.

### 3.2 Thematic Analysis

Thematic analysis identified micro‐, meso‐, and macro‐level factors influencing help‐seeking for EDs (Table 2). While presented as discrete levels, these factors are dynamic and interdependent, shaping participants' experiences through reciprocal influence. Many themes identified were consistent with previous research (Ali et al. 2016, 2020; Potterton et al. 2020). The following sections elaborate on themes which are novel to this study (Table 2) and further information on non‐novel themes can be found in supplementary information. Quotations have been edited for brevity and clarity.

**TABLE 2:** Summary of barriers and facilitators to help‐seeking.

	Barriers	Facilitators
Micro level	Shame as an internalised barrier*	Social locations that give power*
	Failure to perceive severity of illness	Concerns about physical health
	When letting go feels risky	Other mental health problems
Meso level	Stereotypes as relational barriers to care*	Inclusive and affirming care*
	When physical spaces exclude*^a^	Early intervention
	Unseen and under‐represented*	Social support (family and peers)
	Culturally mediated meanings of help‐seeking*	Positive experiences with healthcare
	Lack of support from others (e.g. GP, family)	
Macro level	Marginalisation of complexity*	Seeking visibility and validation online*
	Impact of the pandemic	
	Structural bottlenecks to help‐seeking	

*Note:* Asterisk indicates novel themes identified in the present study while items without asterisks are consistent with existing research on help‐seeking for ED (Ali et al. 2016; Potterton et al. 2020) (Supporting Information S2).

^a^ Interviews in this study were conducted prior to the UK Supreme Court judgement in April 2025 ruling that ‘sex’ refers to biological sex not a person's gender identity which has ramifications for provision of single‐sex spaces in health care.

### 3.3 Barriers

#### 3.3.1 Micro‐Level (Individual Factors)

##### 3.3.1.1 Shame as an Internalised Barrier

All participants identified stigma or shame as barriers to help‐seeking for EDs. Several described feeling undeserving of care, perceiving others as severely ill and thus more deserving of assistance, or experiencing shame associated with seeking help for mental health problems. Body weight was connected to stereotypes about ED, shame, and minimisation of their ED. These accounts suggest an interaction between micro‐level shame and self‐stigma and meso‐level experiences of stigma from others, which together shaped perceptions of deservingness for care. Feeling both less entitled to support and deserving of blame appeared to compound barriers to help‐seeking and exacerbate delays in accessing treatment.

we've always been under the impression that if you're bigger, it's because you have no control and no willpower … I didn’t know much about binge eating, so I didn’t know I could ask for help for that. I thought that was just me being, you know, greedy … asking for help when I look the way I do just felt really, really embarrassing and like they were going to laugh or judge me or just dismiss me

(Mixed ethnic background, woman, BED)

#### 3.3.2 Meso‐Level (Interpersonal Factors)

##### 3.3.2.1 Stereotypes as Relational Barriers to Care

All participants described knowledge of the prevailing stereotypes of EDs as primarily affecting ‘slim, white, affluent, girls’ (SWAG). Participants perceived themselves as falling outside this stereotype, leading them to minimise their symptoms or to underestimate the severity of the problem. They also felt these stereotypes shaped others' responses, contributing to dismissal of their experiences. This dual effect, on self‐appraisal and interpersonal interactions, significantly impeded help‐seeking behaviours. Not only did it deter participants from seeking help, but it also contributed to delays in recognition and appropriate referral once help‐seeking had commenced.

I think being male, I probably took a while to realise my own eating was disordered, and I think it took the, maybe some of the healthcare people I came across a longer time to, to consider it and then when it was considered … I didn't probably fall into a kind of a classical appearing person who had an eating disorder and so some people were a bit thrown by that

(Man, OSFED)

‬‬All participants reported seeking help from their GP for their ED, making it notable that many felt their symptoms were overlooked at this initial point of professional contact. Prevailing ED stereotypes about body size, ethnicity, and gender shaped GP interactions, contributing to perceptions that their diagnosis was overlooked. These experiences appeared to interact with micro‐level processes of self‐stigma and shame, reinforcing doubts about whether they deserved support.

I feel like that's [ethnicity] a big, significant part of …. the first GP [General Practitioner] I did see was an Asian GP, and he was just like, I don't know, I feel like he just didn't get it because it wasn't something he [sic] ever come in interaction with

(Asian woman, ARFID)‬‬‬‬‬‬‬‬‬‬‬‬‬‬‬‬‬‬‬‬‬‬‬‬‬‬‬‬‬‬‬‬‬‬‬‬‬‬‬‬‬

I have had negative experiences with the GP, though in regard to like my weight and stuff … they would immediately just assume it was a weight loss issue as opposed to … a mental health issue‬

(Mixed ethnic background, man, BED)

##### 3.3.2.2 Unseen and Under‐Represented

Participants described feeling that their experiences were under‐represented in ED resources, treatment, and broader media. When they sought educational materials online about EDs, these reinforced stereotypes about EDs and did not include diverse representations of ethnicity, sexual orientation, and gender identity. Limited representation, both in educational materials and in treatment settings reflected broader macro‐level structures, contributing to feelings of exclusion and reinforcing beliefs that their experiences would not be recognised or understood within ED treatment.

like even in like programs you might watch, it's always … the typical … eating disorder of either anorexic and bulimic experienced by a white person. But like, never any person of colour that has a [sic] eating disorder

(Asian woman, ARFID)

I contacted them, but I kind of thought I'm, I'm not really the demographic. It's young, much younger people than me. It's girls, really. And not, sort of, men of advancing years. I just thought this is probably not the right organisation … for me

(Man, BED)

Many participants felt their marginalised social locations were invisible in healthcare interactions, forcing self‐disclosure during help‐seeking. As a result, participants perceived treatment as not appropriately tailored to their needs, which hindered engagement and, in some cases, deterred them from pursuing further support. This lack of recognition also contributed to feelings of being misunderstood or dismissed by healthcare providers.

I did try to start talking about that [sexual orientation] a little bit, but I was just … told that eating disorders affect how you feel about intimacy and how you feel about relationships and it's, it's probably just that … so I felt like that was quite dismissive

(Transgender man, AN)‬‬‬‬‬‬‬‬‬‬‬‬‬‬‬‬‬‬‬‬‬‬‬‬‬‬‬‬‬‬‬‬‬‬‬‬‬‬‬‬‬

##### 3.3.2.3 Culturally Mediated Meanings of Help‐Seeking

Participants' cultural community was an important factor in influencing help‐seeking behaviour. For participants with multiple marginalised identities, these influences appeared to interact. For example, one Asian man described his experience as shaped by intersecting expectations of masculinity and cultural norms of self‐reliance, both of which discouraged disclosure and help‐seeking.

It's either not spoken about or, when it is, it's very like hush hush or like we don't talk about that thing or that person

(Asian, man, BED)‬‬‬‬‬‬‬‬‬‬‬‬‬‬

The intersection of cultural norms and stereotypes about EDs also shaped recognition, with some participants reporting that symptoms were dismissed by members of their family or community.‬‬‬‬‬‬‬‬‬‬‬‬‬‬‬‬‬‬‬‬‬‬‬‬ Cultural beliefs stigmatising mental health problems intensified feelings of stigma and shame and were barriers to help‐seeking and family support.‬‬‬‬‬‬‬

I’ve always kind of been told … ‘[Asian] people don’t develop eating disorders', they don’t … because there’s not always enough food and people just, they’ve got to be lucky for what they’ve got‬

(Mixed ethnic background, woman, AN)

within, like, cultural environments, it might be like. … shame … like how we don't tell anyone, we'll deal with it within ourselves rather than telling anyone about it because of like, oh, ‘what if your community find out, what your family find out?’ ‘What if other people find out?’. It's embarrassing for us rather than the fact that that person needs support

(Asian, woman, ARFID)‬‬‬‬‬‬‬‬‬‬‬‬‬‬‬‬‬‬‬‬‬

##### 3.3.2.4 When Physical Spaces Exclude

Participants felt treatment spaces lacked accessibility and were not adapted for their needs, for example due to limited facilities for men on inpatient units. Transgender participants reported additional challenges, including uncertainty around bed placement, which delayed care but also contributed to the invalidation of their gender identity and intensified shame as an internalised barrier.

Gender had a massively big impact on my care. As a trans male. You also have the impact of it being, the lack of male facilities within eating disorder care as well, so then that sort of adds to it

(Transgender man, ARFID, AN)

Other men spoke about the gendered nature of ED treatment in the design of local facilities. For example, participants perceived the aesthetic and design of the space as being oriented towards women, contributing to a sense of exclusion for male service users. This reinforced stereotypes about EDs and contributed to participants' internalised stigma, fostering perceptions of being unsuitable for treatment.

all the materials I was given … all female pronouns, all very feminised … loads of information about periods and this kind of stuff and nothing about male sexual function at all, like just completely erased‬

(Man, BN)‬‬‬‬‬‬‬‬‬‬‬‬‬‬‬‬‬‬‬‬‬

Inaccessible clinical environments were experienced as structural barriers, where the lack of accommodation for diverse bodies and disabilities impeded accurate assessment and constrained access to care. Across diverse marginalised social locations, this lack of accessibility was experienced as conveying a broader message that these spaces were not designed with them in mind, implicitly signalling that services were not intended for people like them.

they couldn't weigh me themselves, and it was this big fight. It's just kind of a bit ridiculous that basic care for disabled people was a barrier to accessing treatment for an eating disorder

(Transgender man, ARFID, AN)‬‬‬‬‬‬‬‬‬‬‬‬‬‬‬‬‬‬‬‬‬‬‬‬‬‬‬‬

#### 3.3.3 Macro‐Level (Systemic Factors)

##### 3.3.3.1 Marginalisation of Complexity

Participants described current ED treatment pathways in the UK as insufficiently responsive to the complexity of their needs. They positioned themselves as marginalised by systems that did not readily accommodate their experiences, whether due to diagnoses not routinely commissioned for treatment (e.g., BED, AFRID), co‐occurring conditions, or symptom presentations that did not align neatly with existing diagnostic criteria.

it’s [ARFID] not [treated] under the NHS, so I have to … seek privately funded treatments for that …. that definitely felt like it's not serious enough‬

(Asian woman, ARFID)‬‬‬‬‬‬‬‬‬‬‬‬‬‬‬‬‬‬‬‬‬‬‬‬‬‬‬‬‬‬‬‬‬

I don't really relate to a lot of the ways that eating disorders are defined and described and that kind of stuff. I don't know, I feel quite excluded from a lot of them in terms of even the symptoms

(Man, AN, ARFID)‬‬‬‬‬‬‬‬‬‬‬‬‬‬

Taken together, these accounts suggest a dynamic in which participants experienced themselves as pushed to the margins of care. Attempts to seek help were perceived as met with exclusion, while the misalignment between their experiences and established pathways appeared to undermine the perceived legitimacy of their ED. In this way, structural criteria not only shaped access to services but also influenced how participants understood their own need for support. Some participants with an ARFID diagnosis were directed towards private treatment options, underscoring how diagnosis, service access criteria, and socioeconomic factors intersected to limit care.

Participants described negative experiences related to meeting eligibility criteria for specialist ED treatment. A frequently reported concern was the reliance on Body Mass Index (BMI) thresholds to determine access, with participants recounting being denied treatment on the grounds that their BMI was not sufficiently low. These accounts suggest that exclusion did not simply act as a barrier to help‐seeking, but risked reinforcing the disorder, as participants felt they needed to become ‘unwell enough’ to meet criteria, interacting with micro‐level shame as an internalised barrier.

I think I'm actually finding it harder now … because my BMI is not under 17, I just cannot access any help whatsoever, and it's almost as if I've got to get really underweight before anybody will help with the eating disorder.‬

(Woman, AN)‬‬‬‬‬‬‬‬‬‬‬‬‬‬‬‬‬‬‬‬‬

Participants who experienced co‐occurring physical or mental health conditions described a siloed healthcare system that treated difficulties in isolation, with limited communication between services. Those with complex presentations reported barriers to accessing ED treatment, including exclusion from services due to a personality disorder diagnosis or difficulty accessing gender‐affirming care alongside ED treatment. Some described hiding or minimising symptoms to avoid losing access to treatment. Taken together, these accounts suggest that the structure of health systems marginalised those with complex needs, positioning them as not fully fitting within existing service boundaries. ‬‬‬

gastroenterology will be like, oh it's all in your head kind of thing, so we don't need to see you, you need to go to the eating disorder service, they'll deal with it. But the eating disorder service doesn't have any medical people in it … so it's sort of like being passed around a lot between different people, and a lot of de‐legitimisation of problems

(Man, BN)

I feel like I don’t have a right to be there because I’m not, because I’m not like the other people … they have a proper eating disorder and because mine is … this mixture of crap and it doesn’t make a lot of sense. I just don’t have a right to have any treatment because nobody knows what to do anyway

(Woman, OSFED)‬‬‬‬‬‬‬

### 3.4 Facilitators

Participants identified factors that supported help‐seeking, which were also categorised at the micro‐, meso‐, and macro‐levels using a framework of intersectionality. As with barriers, many facilitators were consistent with previous research and are available in Supporting Information S2.

#### 3.4.1 Micro‐Level (Individual Factors)

##### 3.4.1.1 Social Locations That Give Power

While all participants reported characteristics associated with marginalisation, some social locations conferred relative power that facilitated help‐seeking. Higher SES or education enabled access to private care and supported clearer articulation of needs to providers. Presenting at a lower body weight, more closely aligned with dominant ED stereotypes, not only appeared to validate participants' experiences within services but may also have shaped their own recognition of illness, reducing self‐doubt about whether they deserved help.

I'm able to sort of articulate myself in help seeking and … know where to look … so that comes with some power … you can have all those sort of powerful characteristics

(Man, BN)‬‬‬‬‬‬‬‬‬‬‬‬‬‬‬‬‬‬‬‬‬

#### 3.4.2 Meso‐Level (Interpersonal Factors)

##### 3.4.2.1 Inclusive and Affirming Care

Participants reported that having their identities affirmed facilitated help‐seeking. For Transgender participants, this meant access to gender affirming care or ED specialist treatment which embraced their gender identity. These accounts suggest that identity affirmation directly mitigated previously described experiences of invisibility and under‐representation, positioning recognition as not merely supportive but as actively reducing barriers to help‐seeking.

I noticed that as soon as I came out as being trans and I started gender affirming treatment I felt that that was kind of the start, I mean truly letting go of my eating disorder

(Transgender man, AN)

I made a list of things that I find helpful in therapy sessions and things that are maybe not so helpful, and the therapist that I had did try to implement those

(Transgender man, AN)‬‬‬‬‬‬‬‬‬‬‬‬‬‬‬‬‬‬‬‬‬

#### 3.4.3 Macro‐Level (Systemic Factors)

##### 3.4.3.1 Seeking Visibility and Validation Online

Participants described accessing psychoeducational resources about EDs online to better understand the problems they were experiencing. For some, it provided vital information about their unique symptoms (such as ARFID), and they used it to facilitate conversations with primary care clinicians to advocate for referrals to ED specialist services. ‬‬‬‬Content created by people with similar social locations (e.g., ethnic minority background, or gay men) provided validation, normalised experiences, reduced stigma, and ultimately supported formal help‐seeking behaviours. These accounts suggest that when participants did not see themselves reflected in traditional care pathways or service materials, they sought recognition and representation in online spaces. In this way, digital communities appeared to function as alternative sites of visibility and belonging, enabling participants to legitimise their distress and move towards formal support.‬‬‬

I remember reading this kind of like community magazine … they shared an article … with someone South Asian … shared her experiences in an article about her eating disorder … It was very … relatable reading, what she spoke about, and especially from a cultural perspective

(Asian, man, BED)‬‬‬‬‬‬‬‬‬‬‬‬‬‬‬‬‬‬‬‬‬

## 4 ‬‬‬‬‬‬‬‬‬‬‬‬‬‬‬‬Discussion‬‬‬

In this study, an intersectionality lens was used to explore help‐seeking in under‐served groups with EDs. Social locations and structural systems were shown to interact across different levels to create barriers and facilitators to accessing ED care. This reflects a key tenet of intersectionality theory, that individual experiences cannot be accurately understood by isolating discrete influences (Hankivsky 2014) and that social locations are dynamic and socially constructed (Hankivsky et al. 2014). Considering these factors, we have several recommendations, categorised into micro‐, meso‐ and macro‐level interventions, to improve engagement and timely access to ED treatment for people from under‐served groups (Table 3).

**TABLE 3:** Summary of recommendations to improve help‐seeking for ED by people from under‐served groups.

Micro‐level	Awareness building psychoeducation tailored to marginalised groups (e.g. weight stigma, challenging beliefs about self‐reliance)
Meso‐level	Enhanced ED training/education for primary care practitioners
	Accessibility improvements to services
	Physical space (e.g. chairs, functioning lifts)
	Improve representation in written psychoeducational materials
	Increased awareness for care pathways for neurodivergence
Macro‐level	Increased access to early intervention pathways
	Allow self‐referral to specialist ED services
	Remove BMI thresholds for access to treatment
	Commissioning services for all ED diagnoses

### 4.1 Micro‐Level Recommendations

Participants identified stigma and shame as barriers to help‐seeking for EDs, consistent with research across EDs, other mental health conditions, and stigmatised health problems (Ali et al. 2016; Potterton et al. 2020). These accounts suggest that individual‐level experiences of shame may be shaped through interpersonal and community contexts, as participants internalised culturally mediated beliefs about help‐seeking while also responding to anticipated or previously experienced stigma from others. Several awareness building interventions seek to reduce ED‐related stigma and improve understanding of illness severity (Doley et al. 2017) and these could be adapted to reflect the experiences of marginalised groups. For instance, norms emphasising individual responsibility for health can discourage help‐seeking behaviours (Tsong et al. 2023). Interventions addressing culturally‐mediated beliefs around self‐reliance, emotional restraint, or weight‐stigma for people living in large bodies may help address these barriers. However, such approaches primarily target individual‐level processes and do not address meso‐ and macro‐level influences, such as provider bias or limited social support, which intersectional theory highlights must also be addressed to reduce healthcare inequalities.

### 4.2 Meso‐Level Recommendations

Participants in this study described that positive, validating interactions with primary care facilitated greater likelihood of engaging in treatment (Gumz et al. 2023). However, such engagement only supported ED help‐seeking when healthcare providers recognised the potential link to EDs. These findings add to calls for enhanced education and training for GPs, to improve understanding of the diversity of presentations of ED and to strengthen capacity for effective screening (Aouad et al. 2024; Ivancic et al. 2021).

The present study adds to evidence that inclusive and affirming interactions with healthcare professionals can facilitate access to treatment, highlighting how experiences unfold across micro‐, meso‐, and macro‐levels interactions. Drawing on the minority stress model, which posits that anticipated or experienced discrimination linked to marginalised social characteristics can impact help‐seeking (Santoniccolo and Rollè 2024), these accounts illustrate how individual‐level processes (e.g., self‐stigma or feeling dismissed) interact with provider‐level factors (e.g., limited knowledge or stereotyping) to impact help‐seeking.

Accessibility improvements, such as furniture and weighing scales accommodating for all body sizes and abilities and functioning lifts which can be accessed without staff members (Asaria 2025), are an important community‐level step to reduce shame and enhance help‐seeking. Psychoeducational materials should be inclusive of the diverse ethnic backgrounds, gender identities, and sexual orientations of people with EDs. Treatment pathways should be adapted to accommodate for neurodivergence, demonstrated with the effectiveness of the Pathway for Autism (PEACE) (Tchanturia et al. 2020).

### 4.3 Macro Level Recommendations

Systemic factors contribute to the development and maintenance of EDs (Ahuvia and Schleider 2023; Levinson et al. 2024). Many participants identified barriers to accessing specialist treatment including BMI thresholds, lack of commissioning for some ED diagnoses, and long waiting lists. These accounts illustrate how micro‐level factors, such as BMI, interact with meso‐ and macro‐level service criteria to shape both help‐seeking and access to care, despite evidence that weight is not a reliable indicator of ED severity and that all diagnoses carry substantial psychological and physical morbidity (Ali et al. 2025). Early intervention programs, such as First Episode Rapid Early Intervention for EDs (FREED), reduce wait times and improve clinical outcomes, effectively addressing major structural barriers to help‐seeking (Austin et al. 2022; Radunz et al. 2023). Allowing self‐referral into specialist ED programs of all ages would further facilitate help‐seeking for EDs. Removing BMI‐thresholds and commissioning services for all diagnoses (including ARFID) would increase access and bring services in line with clinical guidelines for the treatment of EDs (e.g., NICE) (McEntee et al. 2023).

### 4.4 Strengths and Limitations

Participants represented a diverse range of social locations which cannot be combined into any one group. Given the distinct experiences of participants, experiences of one group (e.g., people in larger bodies) should not be assumed to reflect another. Nonetheless, commonalities in barriers and facilitators to help‐seeking emerged across those historically under‐served in ED research and treatment. Interviews conducted by an outsider researcher (JW) may have influenced how participants shared their experiences of discrimination. As such, the data may reflect not only participants' interpretations of their experiences, but also how they believed those experiences should be presented to a researcher whose social positioning may be perceived as aligned with power in these contexts. To address this limitation, participant validation was undertaken by sharing the manuscript with study participants and inviting their feedback. Finally, the interpretive nature of reflexive thematic analysis limits generalisability, and pragmatic constraints mean that the full diversity of experiences across multiple marginalised social locations may not have been captured.

A key strength was recruiting a diverse group of participants from groups who are under‐served in ED research and treatment. Participants' accounts aligned with prior findings (Ali et al. 2016; Potterton et al. 2020) supporting their validity but also extended knowledge by evidencing unique challenges and facilitators. In so doing, we respond to recent calls for applying an intersectional lens to the experiences of people from under‐served groups in ED (Burke et al. 2020; Levinson et al. 2024).

## 5 Conclusion

Under‐served groups face distinct facilitators and barriers to help‐seeking including internalised stigma, stereotypes, limited inclusivity, and culturally shaped experiences. In response, we propose clinical and policy recommendations to address disparities in help‐seeking. ED services remain chronically underfunded (Viljoen et al. 2024), creating treatment gaps that disproportionately disadvantage these individuals. Structural limitations intersect with biases in diagnostic criteria, which often overlook diverse ED presentations across cultural, socioeconomic, and gendered contexts.

Addressing these inequities requires multilevel systemic change, service commissioning reform and greater clinical awareness and education. Acknowledging the structural contexts shaping help‐seeking for EDs can support more effective engagement with at‐risk groups, improving outcomes for all.

## Ethics Statement

Ethical approval for the study was obtained via the King's College London Research Ethics Committee (Reference: HR/DP‐24/25‐43192).

## Consent

All participants provided written informed consent to participate prior to commencement of the study.

## Conflicts of Interest

The authors declare no conflicts of interest.

## Supporting information


Supporting Information S1

Table S1

## Data Availability

The dataset collected and analysed during the current study is not publicly available as this may violate participant privacy. The corresponding author can be contacted with questions regarding the data set.
