# Supplementary material for: ‘You Can Be Simultaneously Powerful and Marginalised at the Same Time in Different Ways’. An Intersectional Examination of Barriers and Facilitators of Help‐Seeking for Eating Disorders by People From Under‐Served Groups
Source: Eur Eat Disord Rev. 2026 May 29;34(6):1443–53. doi: 10.1002/erv.70131 (PMC13656757; doi:10.1002/erv.70131)
Supplement: Supplementary file 1 [file ERV-34-1443-s001.docx]

**Supplementary Materials**

**Table S1**

Themes consistent with existing evidence on help-seeking for ED

| Theme | Previous Research | Example Quotes |
| --- | --- | --- |
| Barriers | | |
| Micro level | | |
| Failure to perceive severity of illness | (Ali et al., 2016; Potterton et al., 2020) | *I was struggling to admit it to myself, you know, acknowledge that I had a disorder. Never mind, sort of, trying to convince somebody else (*Man OSFED). |
| Achievement | (Ali et al., 2016; Potterton et al., 2020) | *Then it sort of zoned in on the fact that then losing weight was sort of like this thing that I could do. Like it felt like a achievement, sort of thing.‬* (Transgender man, ARFID)‬‬‬‬‬‬‬‬ |
| Emotion Regulation | (Ali et al., 2020; Potterton et al., 2020) | *And with that came eating, that's how my mum used to handle her emotions, so eating was, it was normal to eat when you felt down. And considering I felt down every day, that's why I was eating so much.‬* (Mixed ethnic background, woman, BED).‬‬‬‬‬‬‬‬ |
| Sense of Control | (Potterton et al., 2020) | *And that was a massive area of stress, I think, and something I didn’t have control over. And I think again, the eating patterns helped me regain control.* (Man, OSFED)‬‬‬‬‬‬‬‬ |
| Macro level |  |  |
| Impact of the Pandemic | (Gallagher et al., 2025) | *I went to the GP in July 2021, it was, we’d been putting it off though. Well, I’d been putting off going. I was kind of trying to deny my problems during lockdown*.‬ (Mixed ethnic background, woman, AN).‬‬‬‬‬‬‬‬ |
| Facilitators | | |
| Micro level |  |  |
| Concerns about physical health | (Ali et al., 2016; Potterton et al., 2020) | *I had two GP appointments and then I was like, I went to the orthopaedic and saw a consultant about this and then I had some private physiotherapy, but we didn't bring up, really, anything about my eating and that really. But I see that both issues have been quite like linked.* (Mixed ethnic background, woman, AN). |
| Other MH problems | (Potterton et al., 2020) | *at every stage would have used the depressive symptoms as my door opener and very much come to the eating symptoms later on.….they were usually an adjunct, to the conversation, rather than a focus for most of it.‬* (Man, OSFED).‬‬‬‬‬ |
| Meso Level |  |  |
| Positive Experiences with health care providers | (Potterton et al., 2020) | *The team was very compassionate, so I’d say that was what was also really helpful, that it didn’t feel stigmatising. It didn’t feel like..I was blamed for my difficulties...it was very validating* (Man, AN). |
| Macro level |  |  |
| Early Intervention | (Potterton et al., 2020; Radunz et al., 2023) | *It was all quite quick.‬... I think that probably helped because I was in, I guess, a better mindset about it and that helped me to get motivation to actually go* (Man, AN).‬‬‬‬‬‬‬‬‬‬‬‬‬‬‬‬ |
| Reliance on primary care and an overburdened health service | (Ali et al., 2016; Radunz et al., 2023) | *they had me on a list...I had one online appointment with them. And then I got an email saying that I couldn’t access the service because the waiting list was too long* (43912-07, woman, BED). |

Ali, K., Farrer, L., Fassnacht, D., Gulliver, A., Bauer, S., & Griffiths, K. (2016). Perceived barriers and facilitators towards help-seeking for eating disorders: A systematic review: Perceived Barriers and Facilitators Towards Help-Seeking for ED. *International Journal of Eating Disorders*, *50*. <https://doi.org/10.1002/eat.22598>

Ali, K., Fassnacht, D. B., Farrer, L., Rieger, E., Feldhege, J., Moessner, M., Griffiths, K. M., & Bauer, S. (2020). What prevents young adults from seeking help? Barriers toward help-seeking for eating disorder symptomatology. *Int J Eat Disord*, *53*(6), 894-906. <https://doi.org/10.1002/eat.23266>

Gallagher, L., Hyam, L., Allen, K., & Schmidt, U. (2025). The continuing impact of COVID-19 on eating disorder early intervention services in England: An investigation of referral numbers and presentation characteristics. *European Psychiatry*, *68*(1), e67.

Potterton, R., Austin, A., Allen, K., Lawrence, V., & Schmidt, U. (2020). “I’m not a teenager, I’m 22. Why can’t I snap out of it?”: a qualitative exploration of seeking help for a first-episode eating disorder during emerging adulthood. *Journal of Eating Disorders*, *8*, 1-14.

Radunz, M., Ali, K., & Wade, T. D. (2023). Pathways to improve early intervention for eating disorders: Findings from a systematic review and meta-analysis. *Int J Eat Disord*, *56*(2), 314-330. <https://doi.org/10.1002/eat.23845>
