# Supplementary material for: ‘You Can Be Simultaneously Powerful and Marginalised at the Same Time in Different Ways’. An Intersectional Examination of Barriers and Facilitators of Help‐Seeking for Eating Disorders by People From Under‐Served Groups
Source: Eur Eat Disord Rev. 2026 May 29;34(6):1443–53. doi: 10.1002/erv.70131 (PMC13656757; doi:10.1002/erv.70131)
Supplement: Supplementary file 2 [file ERV-34-1443-s002.docx]

**Supplementary Materials**

**S2.** Semi-structured interview schedule

*Protocol adapted from: Potterton, R., Austin, A., Allen, K., Lawrence, V., & Schmidt, U. (2020). “I’m not a teenager, I’m 22. Why can’t I snap out of it?”: a qualitative exploration of seeking help for a first-episode eating disorder during emerging adulthood. Journal of Eating Disorders, 8, 1-14.*

I’d like to start by showing you the "Wheel of Privilege and Power" (Duckworth & Hankivsky). This tool helps us think about the characteristics we have that might make us more or less likely to face discrimination or marginalisation. Discrimination means being treated in a way that disadvantages you, or where you are treated less well than others. Being marginalised means being ignored or left out by society, and being treated as less important by society. People who are marginalized might not get the same opportunities, respect, or rights as others. This wheel doesn’t include all types of traits that could be marginalized, but it's a good starting point. On the wheel, the closer you are to the centre, the more privilege you have. The further you are from the centre, the more likely you are to face discrimination or exclusion.

1. Can you tell me if there are any characteristics on the wheel which stand out as an important part of your experience?

Probes:

- How do you feel about this part of yourself?
- Do you think that this characteristic has had any impact on your experience of an eating disorder?

2. Broadening out a little, can you tell me about when behaviours or thoughts around food / body started to become a concern for you?

**Probes:**

- Changes noticed
- Feelings towards / understanding of changes
- Life context including any links with discrimination/marginalisation

2. How did things progress?

3. Do you remember a point when you or others close to you realised you needed help for your eating difficulties? When was it? Why then?

4. Can you tell me about your experience of asking for help for your eating difficulties?

**Probes:**

- Life context including any links with discrimination/marginalisation

5.What steps did you take to seek help for your eating difficulties?

6.Were there any things that helped you to get help?

7.What about family and friends, did they play any role in your help-seeking?

8. Do you think that any of the characteristics you identified in the “wheel of power and privilege” impacted your experience of asking for help with your eating difficulties?

9.Did you have any knowledge of eating disorders and who they affect?

**Probes**

- How well do they match with own identities / characteristics?
- Impact on symptoms / help-seeking

10. Any other comments or reflections around your experience of navigating an eating disorder in relation to your characteristics on the wheel of privilege and accessing effective treatment.
